# Supplementary material for: Leadership development among public health officials in Nepal: A grounded theory
Source: PLoS One. 2021 Nov 5;16(11):e0259256. doi: 10.1371/journal.pone.0259256 (PMC8570488; doi:10.1371/journal.pone.0259256)
Supplement: S1 Text — (PDF) [file pone.0259256.s001.pdf]

Interview Questions/Guidelines

Initial questions

1. Your position at office?
2. Your overall role and responsibilities?
3. Your authority?
4. How you choose public health field (both as a part of study and job).
5. How you came to do a governmental job instead of doing other jobs in private and non-governmental sector?
6. Were there any social and cultural factors that increased/decreased your career development as a PH official? Further prompts: social and economic status of family, residence or locality, etc.
7. In relation to cast/ethnicity, have you ever experienced that it influenced your journey to be a PH official or your existing position?
8. In relation to gender, how has it influenced your journey and position? (only for female)
9. Have you ever been inspired by someone in the area that you are working? OR from other area that you are inspired of?
10. What made you inspired by that person?
11. Have you ever tried to be like the person who inspired you? If so, please tell me an event and its consequences in your field.

Specific questions for female participants

1. Being a female, what were the factors that helped and hindered your career?
2. What was your family's gender culture? What you remember the behavior between son and daughter in your family while you were a child?
3. What did you experienced the matter of gender while you were studying at school level, college level and university level?

4. What types of help did you received (in terms of your education and job) from your family before marriage?
5. What types of help did you received (in terms of your education and job) from your husband and his family after marriage?
6. While working as a public health official, what types of facilities that you got, just because of being a female?
7. While working as a public health official, what types of difficulties that you faced, just because of being a female?
8. Have you ever faced any discrimination in your professional development (such as training, transfer, promotion) being a female?
9. To whom you feel comfortable to work: female supervisor or male supervisor, female staff or male staff?
10. What are your views regarding the role of family in developing female's potentialities?
11. What are the reasons that you think regarding the very few numbers of female public health officials in Nepal?

Additional questions based on the previous interviews

1. How do you lead your staff?
2. What difference you experienced between leading and managing people while you are in-charge of a particular project or program?
3. How would you describe your leadership style and/or behaviours?
4. What you think about the important aspects for people who are successful in their life or who have name and fame in their group/organisation?

Extended questions based on the responses from participants to previous questions

1. As you have worked previously in NGOs sector, what differences did you find working there and working in governmental sector? Regarding social interaction. Regarding professional leadership development.
2. What sorts of efforts the government is doing to develop leadership development among public health officials?
3. Have you ever involved in leadership development course or training?
4. What is your view on female leadership inside the MoH?

Triangulation questions based on the findings

1. Public health as an alternative of medicine degree
2. Role of socialization, schooling and caste culture in leadership development
3. Role of political affiliation in leadership enactment
4. Role of extrovert and introvert personality in public health leadership
5. Views on female leadership, issues of system bias in female leadership
6. Role of multitasking in balancing leadership positions among male and female
