# Supplementary material for: Leadership development among public health officials in Nepal: A grounded theory
Source: PLoS One. 2021 Nov 5;16(11):e0259256. doi: 10.1371/journal.pone.0259256 (PMC8570488; doi:10.1371/journal.pone.0259256)
Supplement: S2 Text — (DOCX) [file pone.0259256.s002.docx]

**Memo Writing – Interview No. 11**

It became very difficult to arrange interview time with this participant. I had to wait for 3 weeks to conduct an interview with her. She was very busy, most specifically in the sense that she always keeps herself busy in doing official work and she usually had the habits of accepting the invitation to participate in meetings, group work, workshop and the like.

I remember the first day when I met her for the purpose of providing research relevant information and to arrange a time for interview. I was just telling her about my research objectives and aims, when she started sharing her experiences working in a remote area as a female. She spoke in such a way that it was an interview. She was speaking in such a way that she found a platform to share her experience for which she was proud. I did not obstruct her. When she stopped speaking, I received her consent and arranged a schedule for interview which was revised for three times.

Social setting and culture are important for individuals' career development. However, family culture dominates the social culture. If a family strongly support a girl child in developing her career, then the social contexts are of less value. This participant developed a sense of self-confidence and so-called male personality (strong, brave, and defensive) from her childhood. During her childhood, she preferred herself calling a boy rather than a girl. By considering herself equal to male in existing society, he worked in very remote areas, tackled with the political pressures, and defence with the male stereotypes' bureaucrats. Because of her nature of defencing and tackling, she often missed the invitation in some of the meetings and workshops. However, she was okay with her habits of spoke out without which initiation to change does not occur.

Participant took confidentiality as a very serious matter. Even she provided written consent; she was anxious whether she will be identified by any means. I assured her about the anonymity, confidentiality of audio recording, transcript, and its secure storage. I told her that she will get a transcript via mail and she have the right to edit and revise the interview. This made her comfortable.
