# Supplementary material for: Leadership development among public health officials in Nepal: A grounded theory
Source: PLoS One. 2021 Nov 5;16(11):e0259256. doi: 10.1371/journal.pone.0259256 (PMC8570488; doi:10.1371/journal.pone.0259256)
Supplement: S3 Text — (PDF) [file pone.0259256.s003.pdf]

Example of initial coding

Interview statements (translated)	Initial coding
Yeah…I think family influenced the way I am leading now. Sometimes… I couldn't find an answer for something. For example, staff deliberately do not turn off the electricity when they go outside. Then, again I think…if they have the same mind as me, they would have been in my place…. that's why, I am in my place and they are in their place…… The time when I had grown up… I think that environment made me like this. I have learnt a lot from my family such as how to manage the resources, how to be sensitive while providing services. I remember, we had spare housing in our village in which the nomads and travellers came to eat and sleep there, even the other people in the villages used to recommend those people coming in our house. Like as…I learnt to help since from my childhood, that image is lifelong. I still felt very sad if I heard some clients returning deprived from services. That service (at my village) in childhood and the current service I am doing is different, but service is service, it is about giving to someone who is in need. In that way, I think socialization influenced me and my career. With socialization, the role that my father and mother played, that also influenced me.	Thinking of family<br>Searching for answers<br>Being resource conscious<br>Comparing with other people<br>Remembering family and social environment<br>Managing resources<br>Being sensitive to provide services<br>Helping the needy people<br>Being responsible to clients<br>Realizing the essence of service<br>Influencing role of parents
……I was doing job in a reputed NGO, but later I left there and came to the government system. What I did there did not bring any significant changes in the lives of the community people. Then I thought, if I apply the same efforts that I did in INGO, it can change the whole district. In there, there was neither recognition nor change…. Just the creation of dependency. Then I felt like this is the type of work I should do in the government system. You make a lot of efforts in INGOs, but you can bring change…you can't do anything until the system	Shifting of job<br>Being conscious with change<br>Analysing the efforts with output<br>Preferring independency<br>Expecting recognition<br>Explaining efforts with change<br>Emphasizing for change

(government) turn the switch on to the INGOs. If you really want to bring change in the community, you should be in government.	

Example of focused coding

Interview statements (translated)	Focused coding
Yeah…I think family influenced the way I am leading now. Sometimes… I couldn't find answer of something. For example, staff deliberately do not turn off the electricity when they go outside. Then, again I think…if they have the same mind as me, they would have been in my place…. that's why, I am in my place and they are in their place…… The time when I had grown up… I think that environment made me like this. I have learnt a lot from my family like as how to manage the resources, how to be sensitive while providing services. I remember, we had spare housing in our village in which the nomads and travellers came to eat and sleep there, even the other people in the villagers used to recommend those people coming in our house. Like as…I learnt to help since from my childhood, that image is lifelong.	Resource consciousness<br><br>Responsibility towards services<br><br>Effects of socialization<br><br>Role of parents
……I was doing job in a reputed INGO, but later I left there and came to governmental system. What I did there did not bring any significant changes in the lives of the community people. Then I thought, if I applies the same efforts that I did in INGO, it can change the whole district. In there, there was neither recognition nor change…. Just the creation of dependency. Then I felt like this type of work I should do in governmental system. You do a lot of efforts in INGOs, but you can bring change…you can't do anything until the system (governmental) turn the switch on to the INGOs. If you really want to bring change in the community, you should be in government.	Passion for change<br><br>Desire for independency and recognition
